# Druggists’ Orders

**Published:** 1887-08

**Authors:** 


### Druggists' Orders.

A Cambridgeport druggist has made a practice for some years of saving in a scrap-book some of the most peculiar orders which he receives. "We are asked for some rather strange things," he said to the writer, " but we can generally guess what is wanted. Many people expect a druggist to prescribe for their ailments, as it saves physicians' charges; and the diagnoses of complaints which come to us are often amusing. Look at these: 'Send me some of the essence you put people to sleep with when you cut their fingers off.' That evidently means ether. 'I want something to take tobacco out of my mouth.' Of course, the scent of tobacco was the thing objected to. 'Send me a baby's top to a nursing bottle, ' means, without doubt, a nursing-bottle top. 'An ounce of the smelling stuff that goes through your brain.' describes very well the effect of inhaling ammonia. 'Something for a sore baby's eye,' is not easy to mistake, though stated rather oddly. Here is a startling order for 'enough epicac to throw up a girl 4 years old.' We cannot help sympathizing with this person, who asks for 'enough anise seed to take the twist out of a dose of senna.' Here is a graphic description of a certain ailment in a request for a 'plaster for a man kilt with stitches.' Perhaps the one who wrote this order for 'something for a caustic woman,' built better than he knew. Here is a request for 'something to knock a cold out of an old woman.' The next one seems to be in hard conditon. She desires 'something for a woman with a bad cough and cannot cough.' No druggist would hesitate for a minute to fill this order: 'Something, I forget the name, but it is for a cure.' 'Our own preparation' will just fill the bill in such a case. But what would we send for 'a swelled woman's foot,' 'a man with a dry spit on him,' and 'a woman whose appetite is loose on her.'"
—*Exchange.*

